# Thymoma Presenting as a Pleural-Based Mass

**DOI:** 10.7759/cureus.12901

**Published:** 2021-01-25

**Authors:** Farhan A Shah, Nelson Greene, Cemil Purut

**Affiliations:** 1 Internal Medicine, Lewis Gale Medical Center, Salem, USA; 2 Pulmonary and Critical Care, Lewis Gale Medical Center, Salem, USA; 3 Cardiothoracic Surgery, Lewis Gale Medical Center, Salem, USA

**Keywords:** thymoma, mediastinal mass

## Abstract

We present a unique case of a satellite pleural-based thymoma. The patient is a 66-year-old Caucasian female with a history of a left pericardial soft tissue mass. She had been asymptomatic. Chest radiograph incidentally revealed an acute increase in the size of the mass. CT scan identified a 5.6 X 5.2 X 4.2 cm mediastinal mass in the left infrahilar region along the left lateral pericardium. Positron emission tomography (PET) scan showed the mass had an increased F18 FDG uptake with standardized uptake value (SUV) of 7.2. Left thoracotomy resected a 81g, 6 X 5.5 X 5.0 cm tan-pink well-encapsulated pedunculated mass displacing the left phrenic nerve. The mass was under the parietal pleura and not attached to the pericardium. Immunohistochemical profile identified the tumor as a thymoma, B1 type. Thymomas are relatively rare in the United States, pleural-based thymomas even more so. Early detection of thymomas is critical to avoid late-stage growths. Pericardial involvement of thymomas increases risk of pericardial effusion, tamponade and a complicated thymectomy. Pleural-based thymomas can result in diaphragmatic paralysis secondary to phrenic nerve involvement.

## Introduction

A thymoma is a neoplasm of the thymic epithelial cells that coordinate T-cell maturation, playing a role in adaptive immunity [1]. Thymomas are often seen with autoimmune conditions including myasthenia gravis, pernicious anemia, and others [1]. Classically, thymomas present in the superior anterior mediastinum and appear as a well-circumscribed and lobulated tumorous mass encapsulated by a fibrous capsule [2]. We discuss a rare case of a thymoma masquerading as a pleural mass abutting the left lateral border of the pericardium.

## Case presentation

**Figure 1:**
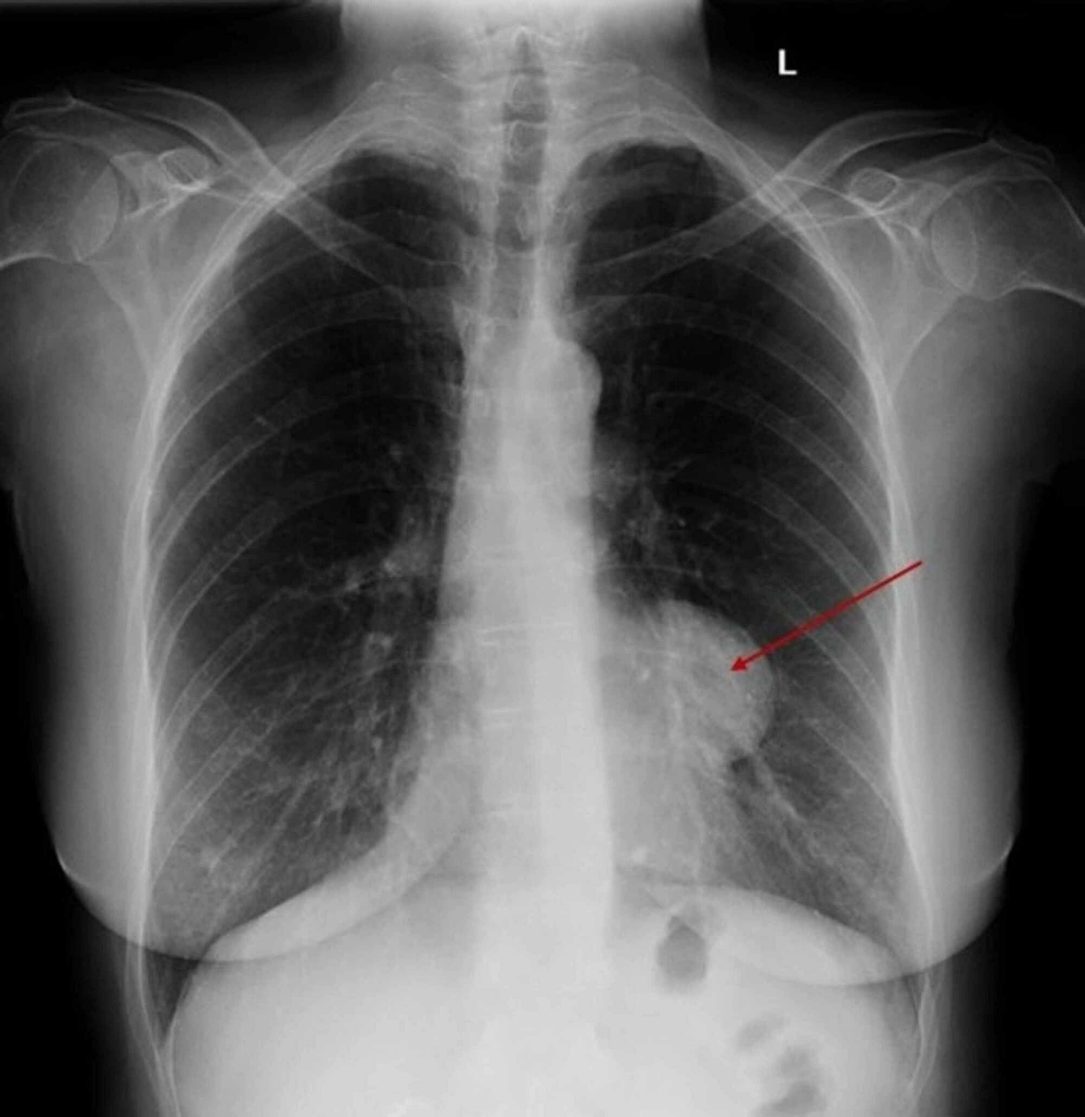
Chest radiograph displaying an anterior mediastinal mass along the left border of the heart.

The patient is a 66-year-old Caucasian female with no significant past medical history except a left pericardial soft tissue mass documented by serial CT scans since six years ago, originally thought to be a pericardial cyst. She had been asymptomatic. A routine chest X-ray completed in preparation for a colonoscopy indicated a gross increase in size of the mass when compared to a radiograph done four years ago (Figure 1). 

**Figure 2:**
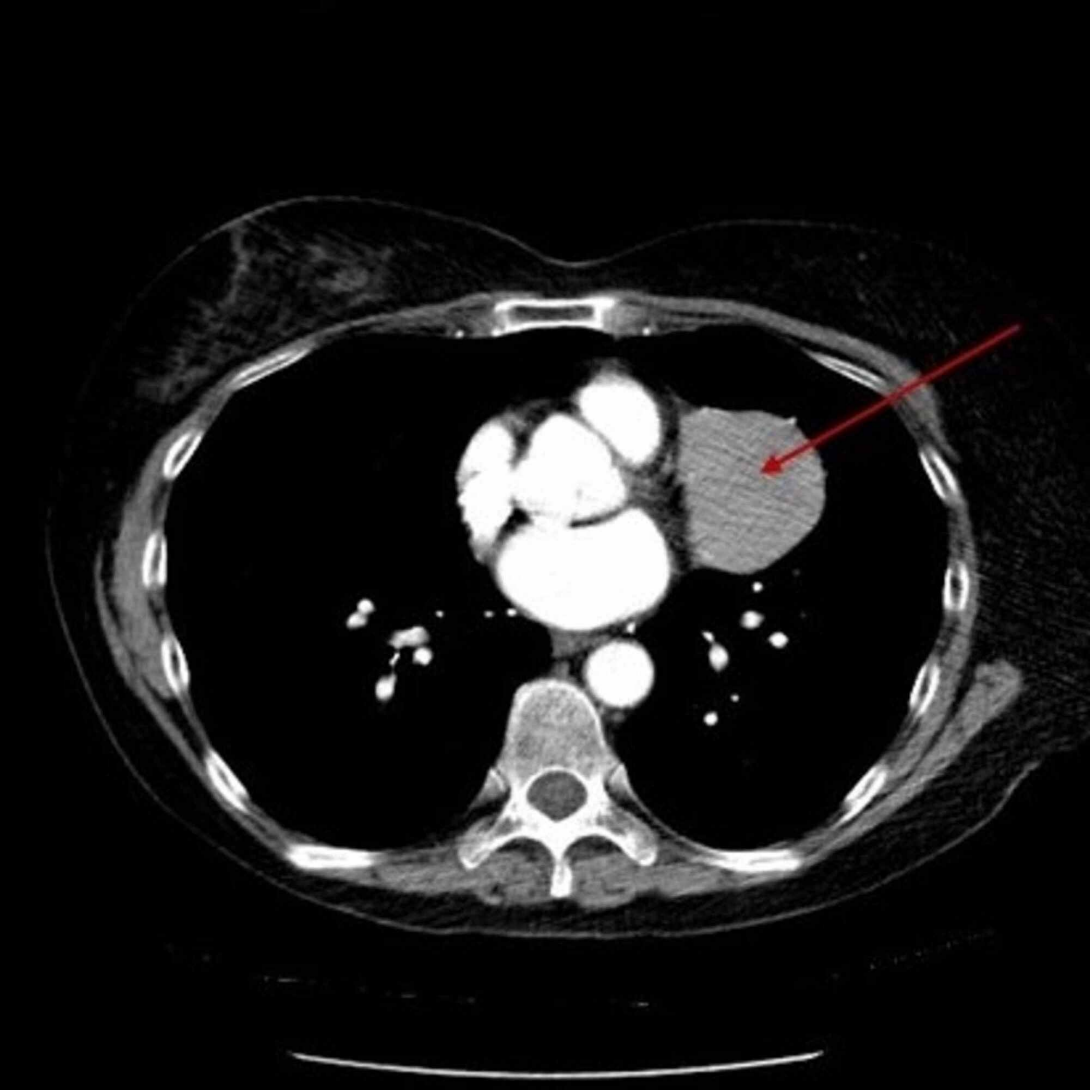
Chest CT scan illustrated a 5.6 X 5.2 X 4.2 cm mass abutting the left lateral pericardium adjacent to the left atrial appendage.

Resulting work-up included a CT scan with contrast, PET CT scan and echocardiogram. The CT with contrast was compared to previous CT scans performed as far back as 2013 and revealed a presence of a mediastinal mass in the left infrahilar region along the left lateral pericardium adjacent to the left atrial appendage with an increase in size from 4.6 X 3.7 X 3.2 cm three years ago to 5.6 X 5.2 X 4.2 cm (Figure 2). 

**Figure 3:**
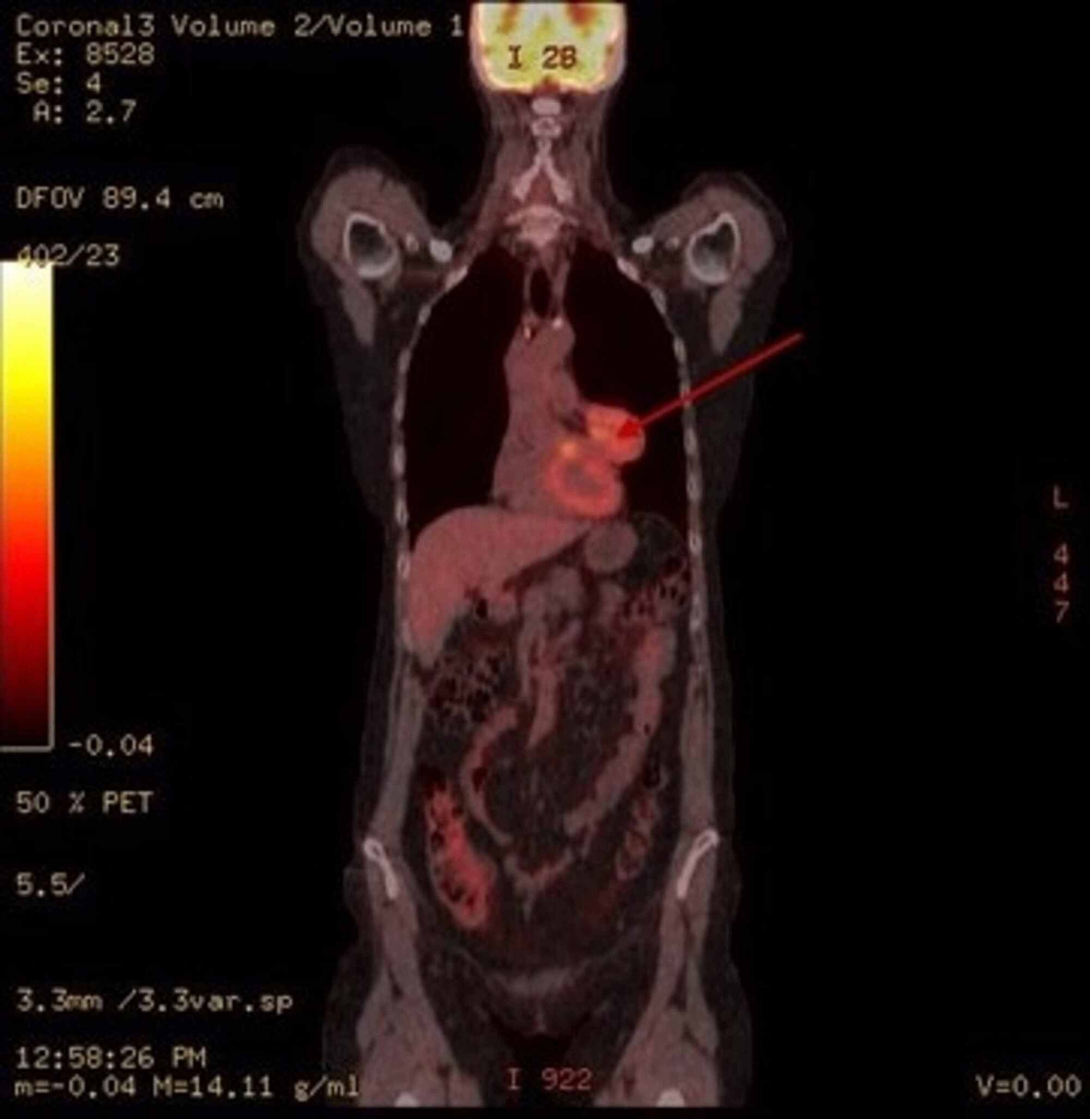
PET scan indicated the anterior mediastinal mass had an increased F18 FDG uptake with SUV of 7.2. SUV: standardized uptake value; PET: positron emission tomography.

The PET CT showed the mediastinal mass had an increased F18 FDG uptake with standardized uptake value (SUV) of 7.2 and multiple calcified and noncalcified mediastinal and bilateral hilar lymph nodes measuring up to 9 mm, with low-grade uptake up to 3.5 SUV (Figure 3). Echocardiogram did not reveal pericardial effusion, pericarditis, mass or involvement.

The patient underwent a left thoracotomy with resection of a 81g, 6 x 5.5 x 5.0 cm tan-pink well-encapsulated pedunculated mass displacing the left phrenic nerve. The mass was under the parietal pleura and not attached to the pericardium. Multiple calcified subcarinal and Left hilar lymph nodes were excised. Frozen section revealed lymphoid tissue. The patient was discharged on postoperative day 3 in stable condition. Final pathology report afterwards showed histologic sections revealing an abundance of small lymphocytes with intermixed epithelioid cells with singular, small nucleoli and open chromatin. The pleural nodules were hyalinized. Immunohistochemical profile indicated this tumor is best classified as a thymoma, B1 type.

## Discussion

Thymomas are relatively rare and uncommonly seen in patients in the United States. According to the National Cancer Institute’s Surveillance, Epidemiology, and End Results (SEER) program data, in the United States the overall incidence of thymomas is 0.13 per 100,000 person-years, approximately 390 cases per year in the United States [1]. The incidence appears to be similar in both females and males, 0.12 per 100,000 person-years and 0.14 per 100,000 person-years, respectively [1].

Thymic epithelial neoplasms have been traditionally classified under the World Health Organization (WHO) classification system [2]. It is divided based on the morphological shape of the thymic neoplastic epithelial cells: Type A is characterized by an oval or spindle shape, Type B is characterized by a round or polygonal shape, Type AB is a combination of the two and Type C refers to a tumorous growth showing histological characteristics of malignancy, regardless of the shape of the cells [2]. Type B1 thymomas are primarily cortical-based [3].

Thymomas have been increasingly found in asymptomatic patients as incidental findings on chest computed tomography scans, correlating with increased routine screening for lung cancer. Around 33% of patients documented and diagnosed with a thymoma are found to be asymptomatic at the time of presentation [4]. While 40% of patients have symptoms related to the thymoma’s mass effect, including cough, dyspnea and chest pain [4]. A well-documented relationship exists between thymomas and autoimmune disorders such as myasthenia gravis, acquired pure red cell aplasia, hypogammaglobulinemia, polymyositis, and Lupus erythematosus. Strikingly, 30% to 50% of patients with a thymoma concurrently have myasthenia gravis, while 10% to 15% of patients with myasthenia gravis have an accompanying thymoma [5].

Differentiating between thymic hyperplasia and thymomas is critical. Contrary to a thymoma, thymic hyperplasia is typically seen on computed tomography as an overall enlarged and symmetric thymus with regular, smooth borders with an intact normal thymic anatomy. A thymoma usually presents as a 1-10 cm mass that is lobulated or smooth and typically arises from a single lobe of the thymus [5]. With increasing size and in the later stages of growth, they will develop irregularity and calcifications. Radiographs may even show elevation of the hemidiaphragm as a result of phrenic nerve involvement by the thymoma [5].

Though thymomas are slow-growing neoplasms, they can become invasively aggressive and invade adjacent structures and involve local vasculature, the pericardium and pleura [5]. Pleural involvement (also known as drop metastases), as seen in our case, can be seen on CT revealing diffuse, nodular or smooth pleural nodules that are ipsilateral to the tumor [5]. However, pleural effusion is rare. Pericardial involvement of thymomas is clinically significant, due to the risk of pericardial effusion, tamponade and a more complicated operative course.

18-FDG uptake on PET scan can help further differentiate between thymic hyperplasia, thymoma and thymic carcinoma. Low-risk thymomas (such as WHO class A, AB and B1) and benign tumors have low FDG uptake, with a mean SUVmax <3.2. While, aggressive neoplasms, such as invasive thymomas (WHO class B2 and B3), thymic cancers (WHO type C) have an increased SUVmax>5 [6]. However, it is important to note that increased physiologic FDG uptake by a normal or hyperplastic thymus is relatively common, especially seen in children and in adults less than 40 years old [5].

Thymoma staging is based upon the Masaoka Staging System. Stage I indicates macroscopically complete encapsulation without any microscopically capsular invasion. Stage II indicates macroscopic invasion into the neighboring adipose tissue or mediastinal pleura or microscopic penetration into the capsule. Stage III indicates macroscopic invasion into a surrounding organ such as the lung, great vessels or pericardium. Stage IVa indicates pleural or pericardial dissemination. Stage IVb indicates hematogenous or lymphogenous metastasis [7].

Surgical resection remains the mainstay treatment option for Stage 1 and 2 thymomas, without the need for adjuvant radiotherapy. Given the minimal tumor remnants seen after surgical resection for a Stage 3 thymoma due to its invasion into the surrounding tissue, adjuvant therapy such as radiotherapy or chemotherapy is recommended. If conditions for a possible macroscopically complete resection for a Stage 4 thymoma are not completely achievable, any surgical therapy plan should be avoided due to the fact that extended resections for Stage 3 and 4 thymomas can be technically demanding and have a high associated morbidity [8].

## Conclusions

Thymomas are relatively rare in the United States, pleural-based thymomas even more so. Early detection of thymomas is critical to avoid late-stage growths. As this case demonstrates, clinicians should be cognizant of the insidious presentation of slow-growing thymomas and treat them accordingly.
